# Social media in surgery: Navigating evidence, sensationalism, and stewardship

**DOI:** 10.1016/j.xjon.2025.11.015

**Published:** 2025-11-24

**Authors:** Dena G. Shehata, Hee-Jung J. Kim, Zhuxuan Pan, Ammara A. Watkins, Elliot L. Servais

**Affiliations:** a Division of Thoracic and Cardiovascular Surgery, Lahey Hospital and Medical Center, Burlington, Mass; b Department of Surgery, UMass Chan Medical School, Worcester, Mass

**Keywords:** social media, thoracic surgery, misinformation, professionalism

**Figure:**
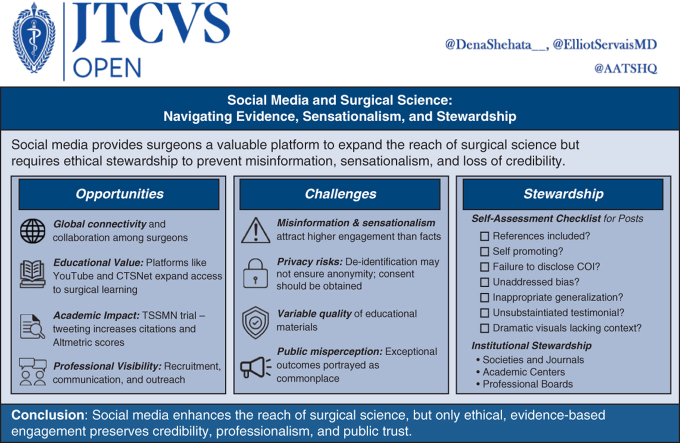

**Figure:**
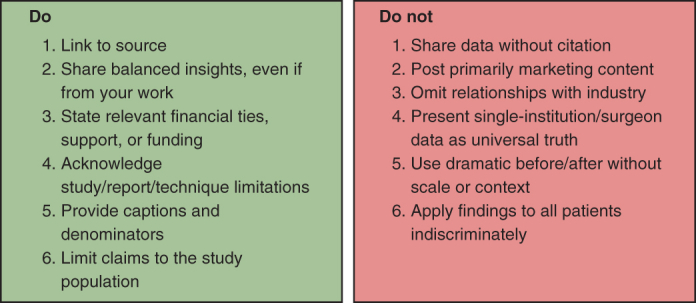
Do's and Don'ts for responsible evidence sharing.

**Central Message**

Social media provides surgeons a valuable platform to expand the reach of surgical science but requires ethical stewardship to prevent misinformation, sensationalism, and loss of credibility.

**Perspective**

Surgeons must balance innovation and visibility on social media with evidence, professionalism, and self-regulation to safeguard trust and academic integrity.

The integration of digital media into academic medicine began in the early 2000s, when physicians started using blogs and online forums to share case studies, research findings, and professional insights beyond traditional academic publishing formats.^1^ With the advent of social media (SoMe), these efforts evolved into a multimodal communication ecosystem, enabling physicians to network, promote clinical practices, and disseminate a wide range of content (Figure 1). Today, nearly 90% of physicians use some form of SoMe for personal purposes, and approximately 65% engage with these platforms professionally.^1^

**Figure 1:**
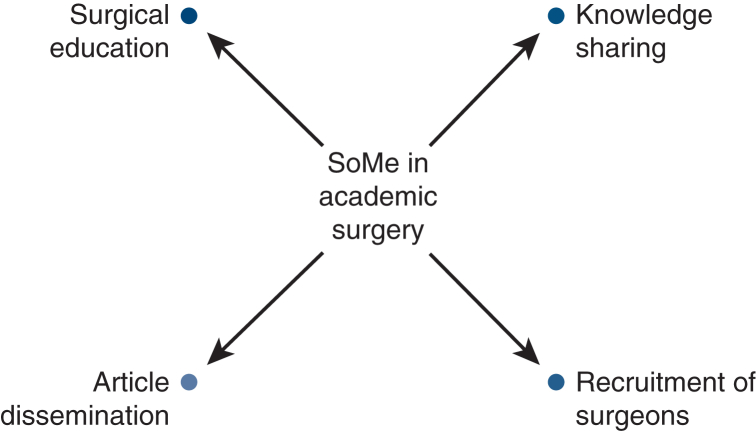
The role of social media (*SoMe*) in academic surgery.

A variety of platforms serve different professional needs, including X (formerly Twitter), Facebook, YouTube, LinkedIn, and ResearchGate. Among these, X has emerged as the most widely used SoMe platform in the health care field.^2^ Its relevance increased during the COVID-19 pandemic, when residency programs created official accounts to improve outreach and communication with prospective applicants.^3^ Among X's defining features is its use of hashtags, which make content easily searchable and accessible to broader audiences. For instance, the hashtag #SoMe4Surgery was used 323,268 times between July 2018 and December 2020, helping surgeons connect, collaborate, and engage in academic discourse across geographic and hierarchical boundaries.^4^ Additionally, the term *Twimpact factor* was coined to reflect the early engagement and citation of articles shared on X.^5^

YouTube is another widely utilized platform, especially for educational purposes. It attracts a diverse audience, including physicians, trainees, medical students, and even patients. In 2020, 40.8% of US adults reported watching health-related videos on YouTube.^6^ Trainees and students often turn to the platform to learn procedural techniques and complex anatomy, whereas patients use it to better understand their diagnoses, treatment options, and prognoses. The rise in engagement and ease of access could serve as an important avenue for advancing public health literacy.

The implications of social media are particularly profound in the field of thoracic surgery. The technical complexity, use of visual learning, and rapid advancements in thoracic surgery make SoMe a valuable tool for education, collaboration, and dissemination. Platforms such as X and The Cardiothoracic Surgery Network (CTSNet) allow thoracic surgeons to share operative techniques and engage in learning on a global scale. However, this increasing social media presence also raises concerns about regulatory oversight, content accuracy, and professional stewardship. As SoMe continues to influence how thoracic surgery is taught, perceived, and practiced, it is crucial to establish a framework that balances these innovations with responsibility. We aimed to explore the influence of SoMe in thoracic surgery, its pitfalls, and various considerations to ensure the responsible use of SoMe within thoracic surgery.

## SoMe in Thoracic Surgery

Building on the broader trends seen in academic medicine, SoMe has emerged as a powerful and increasingly routine tool used in thoracic surgical practice.^7^ The use of SoMe in thoracic surgery gained momentum in the early 2010s, with its initial focus on live coverage of major academic meetings, sharing key takeaways among surgeons and researchers and linking each other to new publications in real time.^8^ The first coordinated thoracic surgery SoMe initiative was the launch of the Thoracic Surgery Social Media Network in 2014, which was a collaborative initiative by *The Annals of Thoracic Surgery* and *The Journal of Thoracic and Cardiovascular Surgery* to promote scholarly discussion and amplify high-impact publications.^9^ In a prospective randomized trial, the influence of this initiative was studied and showed that articles tweeted by the Thoracic Surgery Social Media Network achieved significantly greater increases in alternative metric scores and citations at 1 year compared with nontweeted controls.^10^ These findings highlight that SoMe can serve as an important adjunct for thoracic surgeons, not only to amplify scholarly work, but also to remain engaged, share best practices, and potentially help counter misinformation.

Recent data from a 2025 cross-sectional analysis of American Association for Thoracic Surgery members show that SoMe use among its members was at 42.6%.^8^ X is the most popular platform (32.7% of surgeons, 17.9% active), followed by Facebook (9.4%) and Instagram (7.2%). Professional networking sites such as LinkedIn (78.9%) and CTSNet (98.2%) dominate the online landscape. These platforms serve different functions among the thoracic surgery community to advance academic and clinical objectives. Specialty-specific platforms such as CTSNet host case information dedicated to thoracic surgery, such as videos, tutorials, and forums. The same study also showed that platform use does not differ significantly between adult cardiac, general thoracic, and congenital/pediatric surgeons, but varies by sex, geography, and career stage.^8^ According to the study, female surgeons are more likely to use Facebook and Instagram, and early-career surgeons have higher overall online presence and greater activity on X.

## Pitfalls of SoMe in Academic Medicine and Thoracic Surgery

Despite its advantages, SoMe carries significant risks in both academic medicine and thoracic surgery. A major concern is patient confidentiality: the sharing of images and videos, particularly without informed consent or proper anonymization, creates the potential for serious breaches of privacy.^11^ Before the age of SoMe, most surgical images and videos were shared at medical conferences or literature, at which patient confidentiality was monitored and maintained under the supervision of these platforms with audience limited to medical professionals.^12^ However, SoMe reaches beyond the medical community, catching the eyes of the public, enabling posts with unique stories to garner likes, followers, and attention. In 2023, a study showed that accounts with tweets breaching medical confidentiality had significantly more retweets and followers than medical profiles without.^13^

In addition to the risk of breaching patient confidentiality, the absence of formal quality control mechanisms such as peer review raises concerns regarding accuracy and reliability of shared content. For instance, whereas 84% of sports medicine patients reported using SoMe to seek treatment information, only 47% of Pediatric Orthopaedic Society of North America members maintained active profiles, underscoring the gap between information seekers and qualified professional sources.^14^ Similarly, an analysis of radiology content on TikTok revealed that only 9 of 100 videos reviewed were educational, with the majority consisting of lifestyle or humor-based content.^15^

Furthermore, SoMe enables rapid and unmoderated dissemination of content, which is particularly susceptible to the spread of misinformation. A systematic review of oncology-related posts with high engagement demonstrated that 32.5% contained misinformation and 30.5% contained potentially harmful information, and such posts were more likely to generate user engagement than factual content.^4^ In thoracic surgery, misinformation may present overstated benefits of procedures, minimization of risks, or the portrayal of exceptional outcomes as commonplace.^16^ Beyond public perception, such inaccuracies also threaten the quality of surgical education. A study evaluating 160 laparoscopic cholecystectomy videos found that only 0.06% achieved a passing critical view of safety (CVS) score ≥5, and fewer than 25% reached a CVS score ≥4. Moreover, video popularity (measured by views, likes, or subscribers), did not correlate with educational quality because no significant differences in CVS or global objective assessment of laparoscopic skills scores were noted between highly viewed and less viewed videos.^17^

A more recent study evaluating YouTube videos on robot-assisted segmentectomy found that despite the higher production quality and easier accessibility, most uploaded videos lacked essential educational parameters, such as trocar placement and preoperative assessment details, making them inadequate for surgical training.^18^ Similarly, a 2025 content analysis of Chinese TikTok videos on esophageal cancer showed that nearly 90% of posts provided poor or below-average informational quality, with minimal discussion of risk factors and treatment guidance.^19^ These findings emphasize the variability in educational reliability of surgical content persists across platforms, reinforming the need for professional stewardship to ensure that SoMe complements rather than compromises surgical education for trainees and informed decision making for the public.

Although there are no concrete evaluation metrics or standardized frameworks to assess the effectiveness of SoMe interventions, some literature offers insights into possible approaches. One study measured short-term knowledge acquisition through testing after exposure to Instagram Reels in a neuroanatomy course,^20^ whereas a systematic review of continuing professional development for surgeons noted reliance on self-reported knowledge, competence, and performance. Such strategies could be extrapolated from multiple studies in the future to help create a standardized framework for evaluating the true influence of SoMe interventions.^21^

Thoracic surgeons must apply the same ethical and professional standards online as in clinical practice. This may include obtaining informed consent, ensuring appropriate de-identification of patient information, grounding claims in peer-reviewed evidence, and disclosing conflicts of interest. As SoMe audiences extend beyond professional peers to patients and the public, each post must be crafted carefully in tone and phrasing to prevent misinterpretation, misinformation, and erosion of trust in the surgical profession.

## Ethics and Professionalism

SoMe in academic medicine and thoracic surgery can present unique ethical and professional challenges. When disseminating information on SoMe platforms, it is critical to strike an appropriate balance between evidence and engagement. The term *sensationalism* is defined as the use of exciting or shocking stories or language at the expense of accuracy to provoke public interest or excitement.^20^ (The fast-paced, attention-driven nature of SoMe platforms encourages eye-catching presentation, which may inadvertently compromise the integrity of the content.

**Figure 2:**
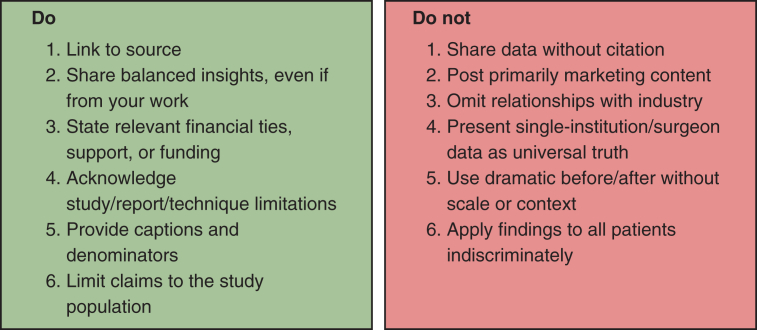
Do's and Don'ts for responsible evidence sharing.

To evaluate whether a post demonstrates elements of sensationalism, several considerations are essential (Figure 2). First, posts should include references that are credible with verifiable sources and clear disclosure of any potential conflicts of interest. Authors must also consider whether the content serves primarily as self-promotion rather than as an objective contribution to knowledge. Additionally, unaddressed biases or inappropriate generalizations can distort the intended message, whereas unsubstantiated testimonial or dramatic visuals without sufficient context may further contribute to sensationalism. Surgeons should self-assess their SoMe contributions by asking whether their posts are supported by evidence (Figure 2), framed in a way that prioritizes education over self-promotion, and consistent with professional standards. Simple reflective questions such as, “Would I present this in an academic forum?” or “Does this add value for patients or colleagues?” can serve as a guide to ensure responsible engagement.

Posts should be evidence-based. The principle of nonmaleficence applies in the digital space: misleading or sensational content can spread rapidly, shaping patient expectations, influencing trainee education, and damaging the credibility of the profession. Professionalism also requires surgeons to avoid behaviors inconsistent with the standards of academic discourse.^21^^,^^22^ Engaging in hostile debates or allowing personal promotion to overshadow educational value undermines the role of surgeons as trusted voices. Maintaining collegiality, transparency, and disclosure of conflicts of interest is essential when using SoMe for academic purposes.

Ethical obligations extend to ensuring accuracy and minimizing harm.^21^^,^^22^ Similar to clinical practice, patient confidentiality and autonomy remain paramount. Sharing clinical images or operative content without informed consent or adequate de-identification risks violating patient rights. Even well-intentioned posts can inadvertently compromise privacy when contextual details or identifiable visuals are overlooked, as de-identification alone may not always be sufficient to ensure anonymity. With patient confidentiality in mind, surgeons should obtain informed consent as best practice whenever feasible, even for content that appears anonymized.

In addition to personal effort, there are institutional strategies to ensure social media contents are evidence-based and checked for sensationalism. An American College of Surgeons committee developed a guideline for ethical use of SoMe by surgeons. The guideline discusses surgeon's patient interaction, role in surgical education, and boundaries between personal online persona and professional representation.^23^ The University of California, San Francisco, provides SoMe training on how surgeons could build digital presence and what academic SoMe posts entail. There is a growing commitment within the surgical community to promote responsible digital engagement while mitigating the risks of misinformation and sensationalism.^24^

## Stewardship

The responsibility for ethical and effective use should extend beyond individual surgeons to professional societies and journals. These organizations can serve as stewards of surgical science and have a unique role in shaping norms, safeguarding accuracy, and promoting evidence-based engagement.

Surgical societies are positioned to establish clear guidelines on the professional use of SoMe, emphasizing confidentiality, evidence-based dissemination, and transparency regarding conflicts of interest. By modeling appropriate conduct and encouraging digital professionalism, societies can help mitigate risks of misinformation, sensationalism, and breaches of trust. Their presence on SoMe also provides a reliable, authoritative counterbalance to the proliferation of unverified sources.^E1^

Journals can also play a complementary role by leveraging SoMe to expand the reach of peer-reviewed research. Active SoMe strategies, such as structured visual abstracts, tweetorials, and moderated journal clubs, enable rapid dissemination of new knowledge to clinicians, trainees, and patients alike. Increasingly, journals are formalizing this role by appointing an associate editor for digital media and scholarship or similar positions on their editorial boards. For example, the *Journal of Thoracic and Cardiovascular Surgery* has both an associate editor for digital media and a Digital Scholarship Committee that meets regularly to guide the American Association for Thoracic Surgery journals’ digital and SoMe strategy. However, this stewardship also entails vigilance; journals must ensure that content posted retains scientific rigor and avoids the dilution of evidence into overly simplified or sensationalized messaging.

Another avenue at the institutional level is for academic centers to formally recognize SoMe scholarship in promotion and tenure processes by requiring structured portfolios, evaluated using scholarly standards (eg, Glassick's criteria) and alternative metrics. In 2016, Mayo Clinic's Academic Appointments and Promotions Committee announced that social media and digital scholarship will be considered for academic advancement.^E2^ Furthermore, a consensus guideline was developed to formerly evaluate digital scholarship and its influence to aid in appropriate appraisal for academic promotion and tenure processes.[Bibr bib27][Bibr bib28] Such approaches legitimize digital engagement while ensuring rigor and accountability.

The broader question remains whether professional boards or certifying bodies (eg, American Board of Thoracic Surgery) should monitor SoMe usage and hold members accountable for misinformation. Although consensus has not been reached, growing recognition exists that unchecked dissemination of inaccurate information online can undermine patient trust and the reputation of the profession.

Raising this point also calls for a discussion of the potential benefits and challenges of such a proposal. On the 1 hand, the potential benefits include enforcing norms of professionalism, deterring harmful behavior (eg, misinformation and undisclosed conflicts), and preserving public trust in the profession. However, there are practical hurdles: such monitoring may threaten academic freedom, lead to effects on discourse, raise privacy concerns, and require resources and clear standards for enforcement. In terms of feasible mechanisms, oversight could take the form of periodic audits, complaint-driven investigations, or integration into existing professional conduct reviews. Consequences for breaches might include graduated responses such as warnings; mandated educational remediation; or in extreme cases, temporary suspension of certification. Any enforcement would need transparent policies, due process protections, appeals pathways, and educational frameworks to avoid perceptions of punitive or suppressive oversight. Although examples and efforts exist,^23^^,^^24^^,^^E4^ the clear definition of roles and the broader applicability of these processes still need to be addressed to streamline implementation.^E5^

Given the dynamic nature of SoMe, with rapid shifts in user behavior and platform features and emergence of new platforms, there is a continuous need to revisit and update guidelines as well as to establish stewardship processes. Ongoing evaluation and adaptation will be essential to ensure that recommendations, when available, remain relevant, effective, and aligned with emerging trends.

## Conflict of Interest Statement

Dr Servais is a consultant for Intuitive Surgical Inc. All other authors reported no conflicts of interest.

The *Journal* policy requires editors and reviewers to disclose conflicts of interest and to decline handling or reviewing manuscripts for which they may have a conflict of interest. The editors and reviewers of this article have no conflicts of interest.
